# Association between [18F]-fluoro-2-deoxyglucose uptake and expressions of hypoxia-induced factor 1α and glucose transporter 1 in non-small cell lung cancer

**DOI:** 10.1186/1749-8090-10-S1-A211

**Published:** 2015-12-16

**Authors:** Takaoki Furukawa, Yoshihiro Miyata, Kei Kushitani, Takahiro Mimae, Yasuhiro Tsutani, Yukio Takeshima, Morihito Okada

**Affiliations:** 1 Department of Surgical Oncology, Research Institute for Radiation Biology and Medicine, Hiroshima University, Hiroshima, Japan; 2 Department of Pathology, Graduate School of Biomedical Sciences, Hiroshima University, Hiroshima, Japan

## Background/Introduction

High maximal standardized uptake values (SUVmax) on [18F]-fluoro-2-deoxyglucose positron emission tomography (FDG-PET) are associated with inferior survival in non-small cell lung cancer (NSCLC).

## Aims/Objectives

Here, we investigated the biological mechanisms underlying FDG uptake in NSCLC.

## Method

This study included 133 patients with NSCLC (109 with adenocarcinoma and 24 with squamous cell carcinoma). The patients underwent tumor resection, at the latest, 4 weeks after FDG-PET. The SUVmax values for primary lesions were calculated based on FDG uptake. The expression of hypoxia-inducible factor 1α (HIF1α) and glucose transporter 1 (GLUT1) was evaluated on immunostained tumor sections using six-point grading scales.

## Results

SUVmax and the expression of HIF1α and GLUT1 were significantly higher in squamous cell carcinoma than in adenocarcinoma (p < 0.001, p = 0.034, and p < 0.001, respectively). In adenocarcinoma, but not squamous cell carcinoma, SUVmax, HIF1α, and GLUT1 correlated with various clinicopathological factors relating to malignancy, and SUVmax and GLUT1 were associated with disease-free survival (DFS) (p < 0.001 and p = 0.029) and overall survival (OS) (p < 0.001 and p = 0.033, respectively). Moreover in adenocarcinoma, HIF1α and GLUT1, GLUT1 and SUVmax, and HIF1α and SUVmax were significantly correlated (p < 0.001 for all), suggesting that HIF1α-induced GLUT1 might influence SUVmax values on FDG-PET.

## Discussion/Conclusion

In lung adenocarcinoma, but not squamous cell carcinoma, HIF1α, and GLUT1 expressions indicate tumor aggressiveness pathologically, and might explain high FDG uptake on PET and correlate with poor prognosis.

